# Central Composite Design (CCD) for the Optimisation of Ethosomal Gel Formulation of *Punica granatum* Extract: In Vitro and In Vivo Evaluations

**DOI:** 10.3390/gels8080511

**Published:** 2022-08-17

**Authors:** Prawez Alam, Faiyaz Shakeel, Ahmed I. Foudah, Sultan Alshehri, Roshan Salfi, Mohammed H. Alqarni, Tariq M. Aljarba

**Affiliations:** 1 Department of Pharmacognosy, College of Pharmacy, Prince Sattam Bin Abdulaziz University, Al-Kharj 11942, Saudi Arabia; 2 Department of Pharmaceutics, College of Pharmacy, King Saud University, Riyadh 11451, Saudi Arabia; 3 Deccan School of Pharmacy, Darussalam, Aghapura, Hyderabad 500001, Telangana, India

**Keywords:** *Punica granatum* extracts, central composite design, ethosome gel, anti-inflammatory action

## Abstract

This research manuscript’s objective was to develop the *Punica granatum* extract ethosome gel. The use of nanotechnology can improve transdermal drug delivery permeation of its major bioactive compound β-sitosterol. The optimised and developed formulations were further studied in vitro and in vivo. The assessment of the anti-inflammatory activity of the gel was performed in Albino rats. Methanolic extract was prepared and developed into an ethosome suspension and an ethosome gel. To optimise the formulation’s response in terms of particle size (nm) and entrapment efficiency (%), the central composite design (CCD) was used in 2^2^ levels. The effects of factors such as lecithin (%) and ethanol (mL) in nine formulations were observed. Characterisation of ethosome gel was performed and the results showed the particle size (516.4 nm) and mean zeta potential (−45.4 mV). Evaluations of the gel formulation were performed. The results were good in terms of pH (7.1), viscosity (32,158 cps), spreadability (31.55 g cm/s), and no grittiness. In an in vitro study, the percentages of β-sitosterol release of ethosome gel (91.83%), suspension (82.74%), and extracts (68.15%) at 279 nm were recorded. The effects of the formulated gel on formalin-induced oedema in Albino rats showed good results in terms of anti-inflammatory activity. The comparative anti-inflammatory activity of *Punica granatum* extract and gel showed that the gel action was good for their topical application.

## 1. Introduction

Globally, in the last decade, the use of herbal products has drastically increased, whether they are food substances, vitamins, or herbal formulations. The major advantages of herbals are fewer side-effects. However, the use of herbal formulations has limitations in the treatment of diseases, which are low oral bioavailability and poor aqueous solubility. These challenges need to be shortened to make them more effective [1]. The scientific literature available for *Punica granatum* peels indicates that they have pharmacology activities [2]; antibacterial [3], anti-coccidial, anthelmintic, antioxidant [4], and antifungal activities [5]; silver nanoparticles for antimicrobial and cytotoxicity effects [6,7]; antitumor properties in different cells [8,9]; activity against multidrug-resistant bacteria [10]; vascular protective effects. The therapeutic effects of herbal medicines are a concern. These may be due to particle size. Bioactive compounds have increased size, which causes difficulty crossing the lipid membrane and, in some cases, the solubility is also a concern. Such causes ultimately affect the bioavailability of the drug. For these reasons, extracts are not clinically used for the treatment of diseases [11,12,13]. The traditional doses of plant extract are converted to a nanoformulation with the application of nanotechnology, which gives several benefits, including enhancement in solubility, permeability, bioavailability, stability, and, ultimately, therapeutic effects [14,15,16]. The nanoformulations’ major achievements are high bioavailability and novel drug delivery [16,17,18].

In pharmaceutical nanoformulation development, the application of Quality by Design (QbD) to optimise the relationship between independent and dependent variables using one-way analysis of variance (ANOVA) statistics is good in terms of optimisation, which gives a robust and economic method. With this approach, it is easy to interpret the variable effects and design the experiments [19,20,21,22]. This research was designed on the basis of existing literature; to date, drug bioavailability has been a concern of research. The goal of this study was to improve drug release by incorporating the extract’s ethosome gel. The major bioactive compound of *P. granatum* such as β-sitosterol was studied in the extract. The result of the study was shown to be better as compared to the different experimental dosage forms. Finally, the therapeutic effects of this formulation are better.

## 2. Results

### 2.1. Optimisation of Formulation

The central composite design (CCD) was used to find the suitable variables. The selection of independent variables is indicated in Table 1. With the selected levels, a total of nine experimental runs were executed, and the recorded results are represented in Table 2. The application of the CCD to establish the relationship between the independent and dependent variables and to optimise the formulation led to a total of nine formulations, and the outcomes are summarised in Table 2.

### 2.2. Response Analysis through Polynomial Equations

#### 2.2.1. Effect of Variables on Particle Size

**Figure 1:**
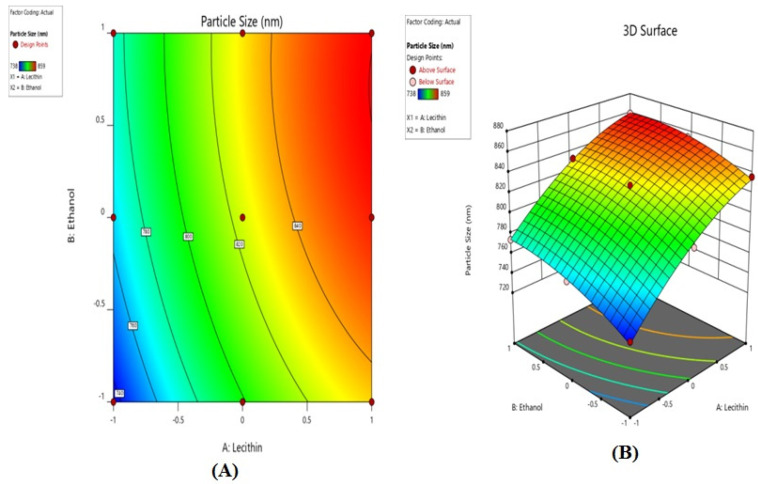
2D contour plots (**A**) and 3D response surface plots (**B**) for evaluating the influence of lecithin (*X*1) and ethanol (*X*2) on particle size (*Y*1).

Data were analysed to fit full second-order quadratic or cubic polynomial equations with added interaction terms to correlate the various studied responses with the examined variables. As depicted in 2D and 3D plots (Figure 1A,B), it is indicated that at lower levels of lecithin, an increase in the levels of ethanol concentration showed a positive influence on particle size. Similarly, by increasing the levels of lecithin, at constant ethanol concentration, an increase in particle size was observed. Thus, the lowest levels of lecithin and ethanol concentrations resulted in a minimum particle size. The final mathematical model in terms of coded factors as determined by the Design Expert software is shown below in Equation (1) for particle size (Table 2).

*Response 1: Particle size, Y*1

*Y*1 = 824.11 + 45.83*X*1 + 16.17*X*2 − 3.25 *X*1*X*2 − 14.17*X*12 − 9.1722 (1)

The ANOVA result is inbuilt; *p*-values less than 0.0500 indicate that model terms are significant. Model terms A, B, and A2 are significant model terms, and a model F-value of 152.43 implies that the model is significant. The values of the predicted determination coefficient (R^2^) and adjusted R^2^ were 0.9631 and 0.9895, respectively, and the differences were less than 0.2. The signal-to-noise ratio value was 34.4816, which indicates an adequate signal.

#### 2.2.2. Effect of Variables on Entrapment Efficiency

**Figure 2:**
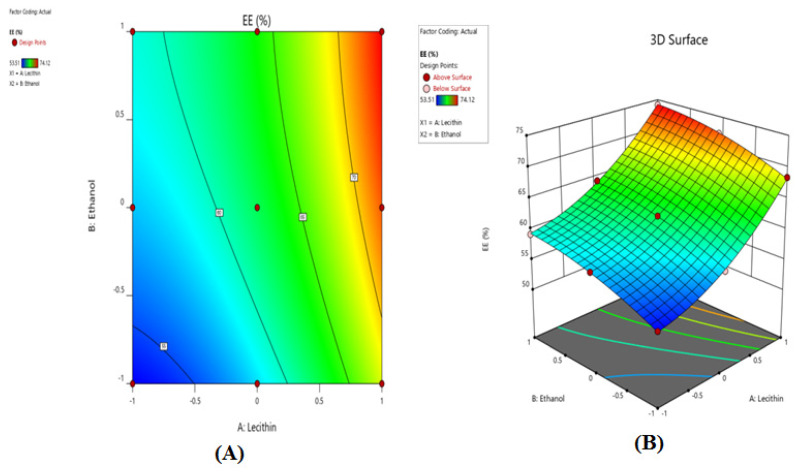
2D contour plots (**A**) and 3D response surface plots (**B**) for evaluating the influence of lecithin (*X*1) and ethanol (*X*2) on entrapment efficiency (*Y*2).

The percent entrapment efficiency of the drug was positively correlated with *X*1, lecithin concentration, and *X*2, ethanol concentrations, as shown by the 2D contour plot and 3D response surface plot (Figure 2A,B). The final mathematical model in terms of coded factors as determined by the Design-Expert software is shown below in Equation (2) for entrapment efficiency (Table 2).

*Response 2: Entrapment Efficiency*, *Y*2

*Y*2 *=* 62.14 *+* 7.41*X*1 *+* 2.99*X*2 *+* 0.0100*X*12 − 1.12*X*22 (2)

The ANOVA result is inbuilt; *p*-values less than 0.0500 indicate that model terms are significant. Model terms A, B, A2, and B2 are significant model terms, and a model F-value of 982.34 implies that the model is significant. The values of the predicted R^2^ and adjusted R^2^ were 0.9926 and 0.9984, respectively, and the differences were less than 0.2. The signal-to-noise ratio value was 89.1626, which indicates an adequate signal.

### 2.3. Gel Punica granatum

#### 2.3.1. Vesicle Size, Particle Size Distribution, and Zeta Potential (ZP)

**Figure 3:**
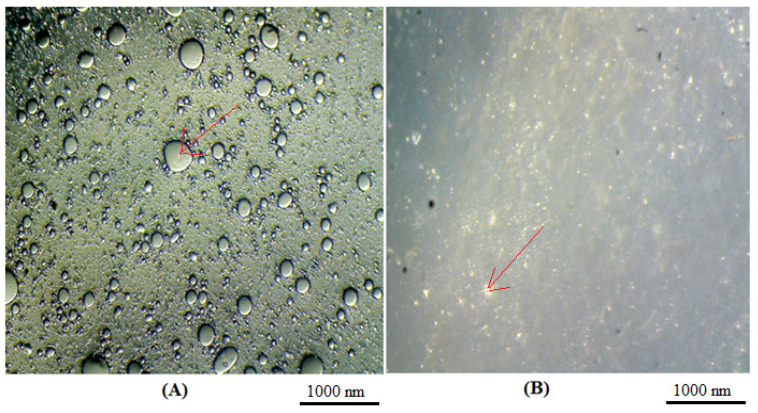
Microscopic view of (**A**) *P. granatum* ethosome suspension and (**B**) *P. granatum* ethosome gel. Arrows show the representation of the respective ethosome suspension and ethosome gel.

**Figure 4:**
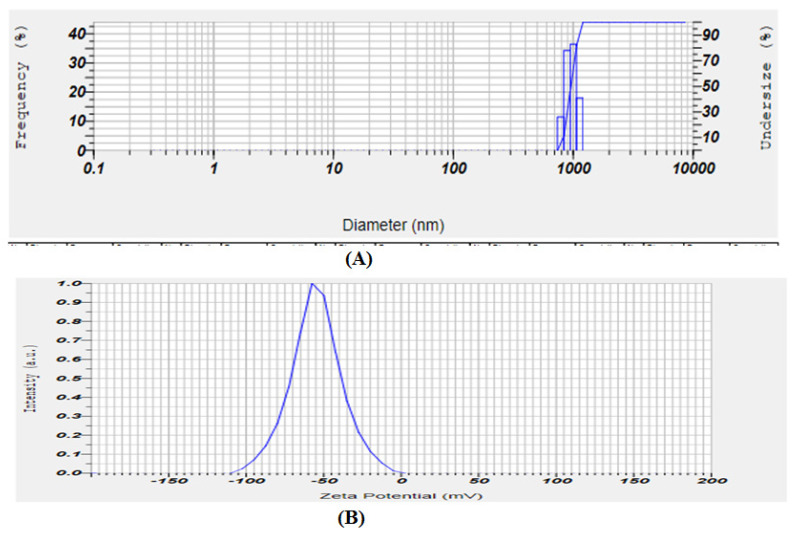
*P. granatum* ethosome particle size (**A**) and zeta potential (**B**) value.

Using various microscope imaging techniques, the generated vesicular formulations were characterised for morphological properties such as vesicle shape, lamellarity, surface morphology, and aggregation. Prior to sonication, the creation of vesicular structures and the shape of the produced vesicles were examined using an optical microscope. The prepared ethosome suspension and ethosome gel were first viewed under the microscope. A drop of diluted vesicular suspension was kept on a clear microscope slide, spread uniformly by putting on the cover slip, and viewed under a 10× magnification optical microscope (Figure 3). The developed ethosome formulation average particle size (516.4 nm) and mean zeta potential (−45.4 mV) values were reported and are presented in Figure 4.

#### 2.3.2. Scanning Electron Microscopy (SEM)

**Figure 5:**
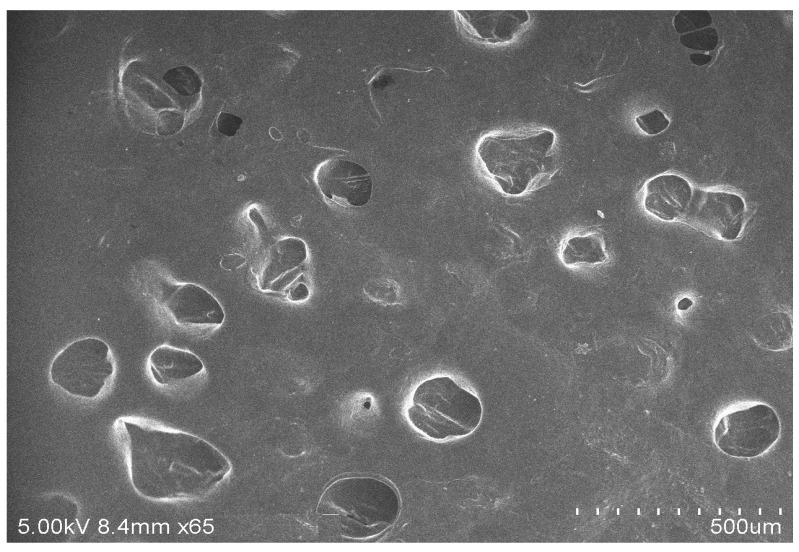
Surface morphology of *P. granatum* ethosome vesicles using a SEM.

The sample was gold-coated with a sputter coater (JEOL, Tokyo, Japan) and examined under SEM with a 20 kV accelerating voltage. The topography of the prepared nanovesicle surface was examined using SEM. The representative SEM image is presented in Figure 5.

#### 2.3.3. FTIR

**Figure 6:**
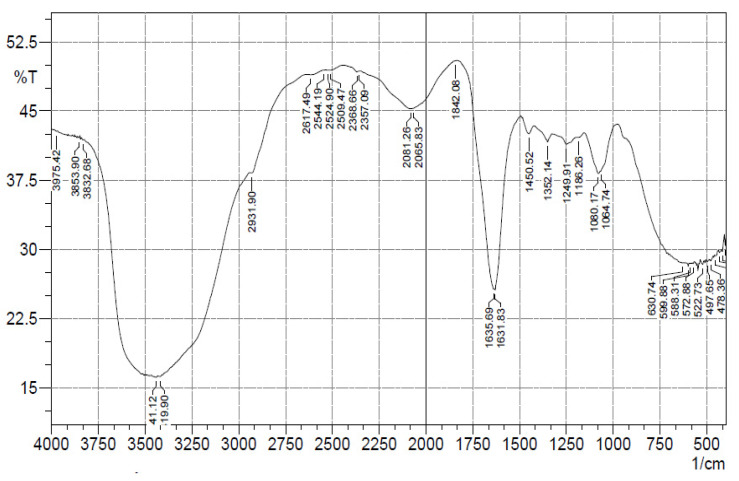
FTIR spectra of *P. granatum* ethosome gel (F9).

Figure 6 shows the result of scanning the *Punica granatum* gel in the FTIR over the fingerprint region (4000–400 cm^−1^). The presence of hydroxyl groups and esters with amide groups in an optimised formulation F9 is shown by the transmission bands at 3500–3200 cm^−1^ and 1700–1600 cm^−1^, respectively. The FTIR of the ethosome gel in this work indicates the existence of both sets of compounds. *Punica granatum* extracts have been described in the literature for numerous types of compounds with hydroxyl, ester, and amide groups. The gel spectrum is only for characterisation and drug release studies at 279 nm in the UV-spectrophotometer, which covers π–π* and n–π* electron transitions, which indicate the presence of such groups in the constituents.

### 2.4. Gel Evaluations

The gel formulation of *P. granatum* extracts was evaluated, and the results of the parameters mentioned are shown in Table 3. The results in terms of pH, viscosity, spreadability, and grittiness indicate the suitability of the gel for its topical application.

### 2.5. In Vitro Diffusion Studies

**Figure 7:**
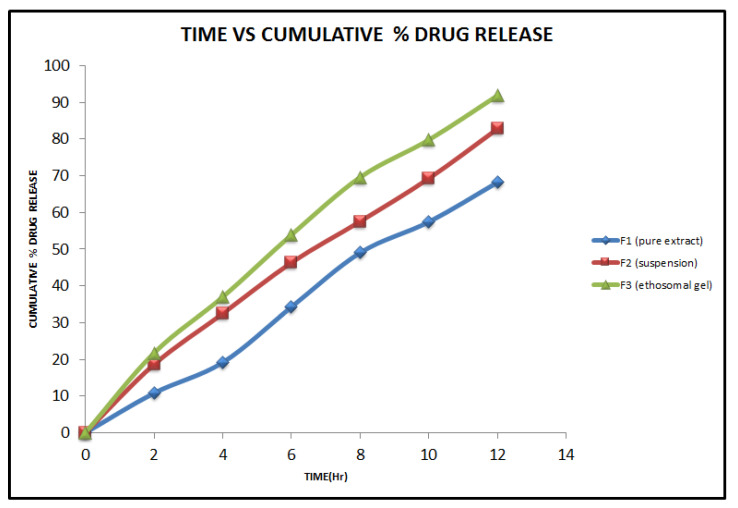
Comparative study of drug release of β-sitosterol from *P. granatum* extract, suspension, and gel.

**Figure 8:**
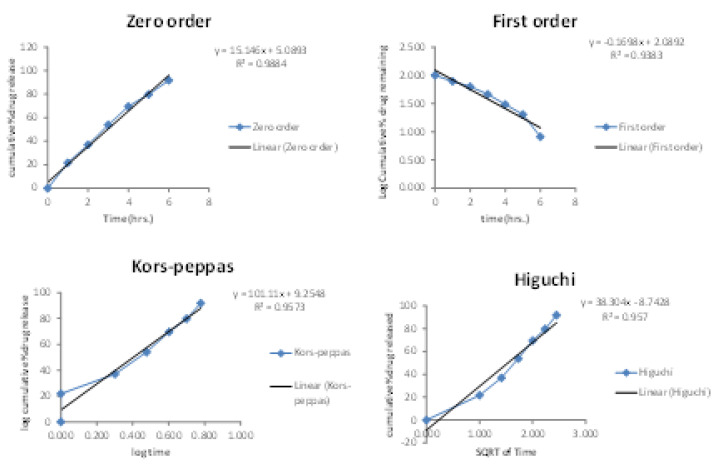
Drug release kinetics of β-sitosterol from optimised formulation F9.

A UV spectrophotometer comparative study of pure extract, suspension, and gel for the drug content was performed and the results are indicated in Figure 7. The results showed that the gel of the extract had better drug content for pharmacological activity. After 12 h, the percentage of drug release of ethosome gel (91.83%), suspension (82.74%), and extracts (68.15%) decreased. The drug release kinetics for an optimised formulation F9 was evaluated by fitting the drug release data with zero-order, first-order, the Higuchi model, and Korsmeyers–Peppas models. The results of drug release kinetics are included in Figure 8. Among the different models studied, the R^2^ value was obtained highest for the zero-order model. Hence, the ethosomal gel formulation followed zero-order kinetics.

### 2.6. Stability Studies

Ethosomal formulations were observed for any change in appearance or colour for a period of 12 weeks. There was no change in the appearance of ethosomal formulations throughout the period of study. The results of characterisation are significant and encouraging, and these were discussed in light of the current concept in Table 4.

### 2.7. Formalin-Induced Rat Paw Oedema

**Figure 9:**
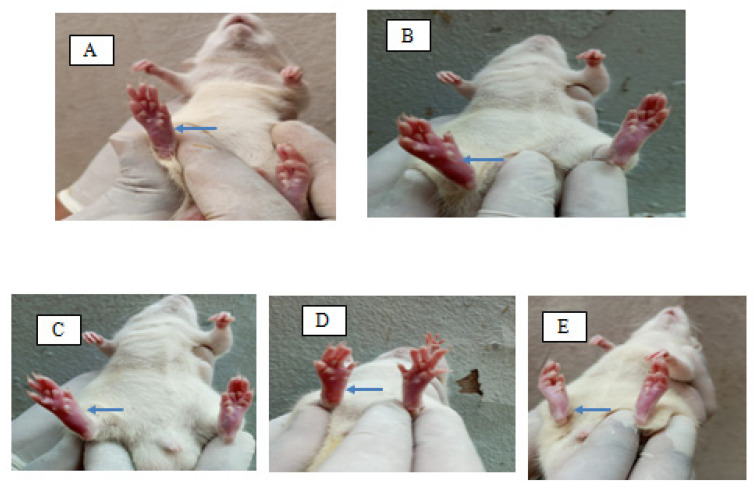
Visual observation of rat during the study of oedema: (**A**) control, (**B**) formalin-induced oedema, (**C**) ethosome suspension, (**D**) gel, and (**E**) standard. Arrow indicates the oedema of each group of rats on the left hind paw.

**Figure 10:**
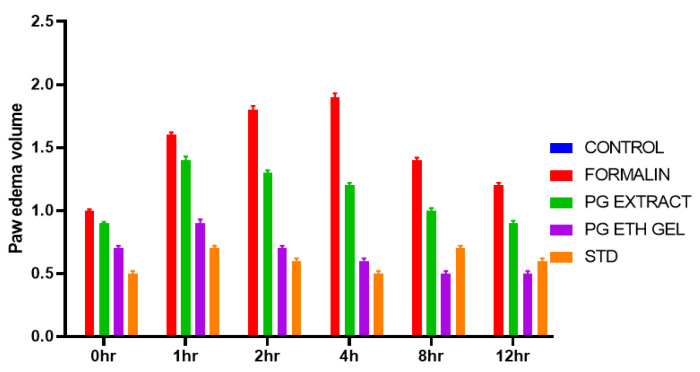
Inflammation volume observation in rats of ethosome suspension and gel.

**Figure 11:**
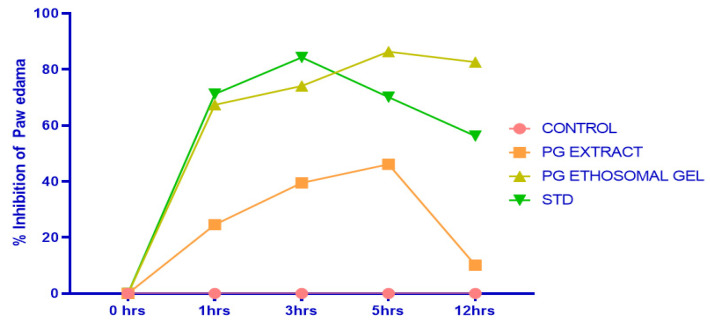
Percent inhibition values of *P. granatum* extract, ethosomal gel, and standard.

In the present study, we assess the reduction in inflammation in rats via formalin-induced rat paw oedema. During the experiment, the visual observation of animals is given in Figure 9. A comparative observation of the results in terms of paw oedema volume compared with time is presented in Figure 10 and Table 5. The % inhibition of oedema with time for the control, extracts, and ethosome gel is shown in Figure 11 and Table 6.

## 3. Discussion

The ethosome gel study on the animal observed the effects of formalin-induced oedema. Methanolic extract of *P. granatum* was used for the preparation of suspension, and after that, the suspension was formulated into gel. Optimisation of the formulation of independent variables and dependent variables was carried out with the QbD approach [23]. The application of QbD with an object was performed to obtain a result that is simple, accurate, and robust. Characterisation of the formulated gels in terms of parameters, zeta potential, particle size, and vesicle size was performed. The results indicated that the developed formulation was good and having acceptable physicochemical properties in terms of pH, viscosity, spreadability, and absence of grittiness [24]. Furthermore, the study was performed to evaluate the ethosome gel formulation in terms of viscosity, spreadability, and pH. All these parameters such as viscosity, spreadability, and pH were found to be suitable for the medical application of developed ethosome gel [25]. In a UV spectrophotometer, a comparison of *P. granatum* suspension and gel was performed for the determination of its major bioactive compound, β-sitosterol. In vitro diffusion studies indicated a significant release of β-sitosterol from the ethosome gel formulation compared to *P. granatum* extract and the *P. granatum* suspension formulation. In vitro diffusion studies suggested the great potential of ethosome gel for the transdermal delivery of β-sitosterol. After performing the in vitro studies of the ethosome gel formulation, the pharmacological activity on rats was divided into four groups. In an animal study, a better result of drug release in ethosome gel was observed in order to reduce the oedema. The anti-inflammatory effects of an optimised ethosome gel of *P. granatum* were significant compared with *P. granatum* extract. These results suggested that ethosome gels have great potential for enhanced anti-inflammatory effects of *P. granatum*. Hence, the ethosome gel formulation can be used as a medicated gel for the transdermal potential of *P. granatum*.

## 4. Conclusions

Ethosome gel of *P. granatum* extracts was evaluated for in vitro and in vivo studies. The prepared gel showed nonvesicle size, stability in pH 7.1, and optimum spreadability without grittiness. The nanoformulation’s particle size and entrapment efficiency enhanced the drug bioavailability. The particle size in the range of nanoformulation increased the drug permeations. The pharmacological activity of the gel formulation results was effective to control the inflammation. The reduction in inflammation with time increased in the case of *P. granatum* extracts gel. These results indicate the great potential of *P. granatum* ethosome gel for enhancing its anti-inflammatory activity. Further in vivo studies such as preclinical pharmacokinetics and clinical investigations are required to explore the complete potential of *P. granatum* ethosome gel for its medical applications.

## 5. Materials and Methods

### 5.1. Plant Material and Chemicals

Polyethylene glycol, lecithin, and cholesterol were purchased from Alpha Chemika, (Mumbai, India). Ethanol and chloroform were purchased from Fisher Scientific (Loughborough, UK). Double-distilled water was obtained from the distillation unit.

Fruits of the *Punica granatum Linn.* (common name: pomegranate, family: Punicaceae) were collected in the Hyderabad, Telanagana, India area. Fresh peels were separated from the seeds. The plant was authenticated by Dr. L. Rasingam, Department of Botany, Osmania University (PCOG.H-221). The collected peel material was washed in running tap water and then rinsed with distilled water. The dried peels were subjected to drying at room temperature for about two weeks in the open air. The dried peels were powdered using a mixer grinder and passed through sieve no. 85.

### 5.2. Instrument Details

A microscope (Labomed inverted microscope, TCM 400, St. Louis, MO, USA), scanning electron microscope (JEOL, JSM-6360; Tokyo, Japan), FTIR spectrometer (IR Affinity-1S, Shimadzu, Tokyo, Japan), and LV-Spindles Brookfield viscometer (Middleboro, MA, USA) were used.

### 5.3. Animals

Adult male Wistar rats (9 to 11 weeks old, weighing 180–250 g) were obtained from Mahaveera Enterprises (Hyderabad, India). The animals were housed under standard laboratory conditions at 25 °C with a 12 h light-dark cycle and unlimited access to chow and water. The research protocol was approved by (HKES’S, MTRIPS/IAEC/1948/Re/S/17/18/2021-22).

### 5.4. Preparation of Peel Extract

The dried 150 g powder was subjected to Soxhlet apparatus extraction using methanol for 72 h. The extracts were concentrated in rotary flash evaporators and stored in a refrigerator till further use [26].

### 5.5. Preparation of Ethosomes Suspensions

The ethanolic extract of *Punica granatum* was dissolved separately in a covered vessel at room temperature by vigorous stirring, and polyethylene glycol was added slowly to this mixture and heated to 30 °C at 800 rpm for 10 min. Lecithin and cholesterol were dissolved in ethanol and added to the above mixture. Double-distilled water was added slowly as a fine stream with constant mixing at 800 rpm for 30 min. The mixing was continued for an additional 5 min. The size of the ethosome vesicles can be decreased using sonication or extrusion methods. The ethosome suspension was refrigerated until further use [27,28]. The composition of ethosomes suspensions is listed in Table 7.

### 5.6. Preparation of Ethosomal Gel

Ethosomal vesicles suspensions were incorporated by the dispersion method using carbopol 940 as the dispersing gelling agent. The specified amount of carbopol 934 powder was allowed to swell overnight. Triethanolamine was added drop-by-drop to the neutralised mixture; the optimised ethosomal dispersion was added and mixed properly. Mixing was continued until a transparent gel appeared and preservative was added; the prepared gels were filled into glass containers and stored at 4–8 °C [29,30].

### 5.7. Experimental Design

Statistical evaluation of the experimental design was performed using Design Expert (v.13.0.3.0, Stat-Ease Inc., Minneapolis, MN, USA) software. The most popular response surface method was the CCD. A 3-level design was employed in this study, requiring 9 experiments. Optimisation of the formulation had a great extent of influence on the composition and development method of ethosome. The 2^2^ design for optimisation of the ethosome was employed to study the effects of independent variables selected such as lecithin (*X*1) and ethanol (*X*2) on dependent variables, i.e., particle size (nm) and entrapment efficiency (EE), respectively. The coded levels translated to the experimental units, experimental runs, and their factor combinations considered in the present study are summarised in Table 1. The composition of ethosome formulations is given in Table 8. The significant model was analysed using ANOVA.

### 5.8. Punica granatum Gel Characterisation

#### 5.8.1. Vesicle Size, Particle Size Distribution, and ZP

Using various microscope imaging techniques, the generated vesicular formulations were characterised for morphological properties such as vesicle shape, lamellarity, surface morphology, and aggregation. Prior to sonication, the creation of vesicular structures and the shape of the produced vesicles were examined using an optical microscope (Labomed inverted microscope, TCM 400, St. Louis, MO, USA). A drop of diluted vesicular suspension was maintained on a clear microscope slide, spread uniformly by putting on the cover slip, and viewed under a 10× magnification optical microscope [31,32].

#### 5.8.2. SEM

The surface morphology of the produced nanovesicles was examined using SEM (JEOL, JSM-6360, Tokyo, Japan). A drop of the vesicular formulation was homogeneously placed on a clean glass slide and allowed to air-dry and observed for size and surface morphology [33].

#### 5.8.3. FTIR

This is a method of identifying pharmacological compounds by observing the functional groups present in the compound. Using an FTIR spectrometer (IR Affinity-1S, Shimadzu, Tokyo, Japan), an FTIR spectrum was comprehensively analysed with potassium bromide (KBr), with spectra ranging from 4000 to 400 cm^−1^ [34].

### 5.9. Evaluations of Ethosomal Gel

#### 5.9.1. Viscosity

The viscosity of ethosome gels was measured using an LV-Spindles Brookfield, DV-E viscometer (Middleboro, MA, USA) with spindle no. 64 at rpm 100 and recorded at a regulated temperature of 25 ± 2 °C (in cps) [35].

#### 5.9.2. Spreadability

Two glass slides of 20 cm × 20 cm were selected. The ethosomal gel was placed between the slides. A weight of 100 g was placed on the upper slide to press the gel uniformly and form a thin layer. The time taken for the separation was noted using a stop-clock. The following equation was used for this purpose [36].

S = m l/t(3)

where S—spreadability, m—weight tied to the upper slide, l—length of the glass, and t—time taken in seconds.

#### 5.9.3. pH Measurement

The pH of the ethosome gels was measured with a pH e-pocket-sized digital pH meter.

### 5.10. In Vitro Diffusion Studies

A diffusion study of formulations was carried out using a Franz diffusion cell through a dialysis membrane. The dialysis membrane was soaked in distilled water for 24 h. The Franz diffusion cell contains two compartments: an upper donor and a lower receptor compartment. The receptor compartment was filled with 7.4 phosphate buffer and the donor compartment contained 100 mL of ethosomes on a dialysis membrane with an exposure area of 2 cm^2^ to the receptor medium. The whole assembly was maintained on a magnetic stirrer at 600 rpm for a period of 600 min, and samples were withdrawn at an interval of 1 h for 8 h and replaced with an equal volume of buffer. Samples were appropriately diluted with buffer and analysed for the drug (β-sitosterol) using a UV spectrophotometer at 279 nm [37,38].

### 5.11. Stability Studies

The optimised ethosome gels were subjected to stability studies in lacquered aluminium collapsible tubes stored at three different temperatures, i.e., 4 ± 2 °C, 25 ± 2 °C, and 40 ± 2 °C, for a three-month period and evaluated for appearance, colour, pH, viscosity, and drug content [39,40].

### 5.12. Formalin-Induced Albino Rats Paw Oedema

The Turner method was used to conduct the formalin-induced anti-inflammatory activity. With a sub-plantar injection of 20 μL of freshly produced 2 percent formalin in the hind paw, oedema was generated in the left hind paw of the rats in all groups [41,42]. The right hind-paw was used as a negative control. The plethysmograph method was used on both hind paws of each group of rats to measure the anti-inflammatory activity and compare it to the control and treatment groups [43,44]. This was performed to maintain a constant paw volume. The left and right paw volumes in each group were measured at the beginning (normal paw volume), as well as at 1, 2, 6, 8, and 12 h after inducing inflammation.

The percent inhibition for oedema was calculated using the following equation:

% Edema inhibition = (V_L_ − V_R_) control − (V_L_ − V_R_) treated/(V_L_ − V_R_) control × 100. (4)

where V_L_ is the left paw displacement volume and V_R_ is the right paw displacement volume.

### 5.13. Statistical Analysis of Results

The statistical software for social version 17.0 (SPSS) was used to evaluate the data. Descriptive statistics were employed to show data in terms of mean ± SEM using ANOVA, followed by a post hoc test by using Tukey’s multiple comparison test. The data were analysed using GraphPad Prism software (version 8.4.2; San Diego, CA, USA) [45].

## Figures and Tables

**Table 1:** Selection of independent variables.

Coded Values Level	Independent Variables	
	*X*_1_, Lecithin	*X*_2_, Ethanol
−1	1	15
0	1.5	20
+1	2	30

**Table 2:** Composition and characteristics of formulations.

Formulation Code	*X*_1_, Lecithin	*X*_2_, Ethanol	*Y*_1_, Particle Size (nm)	*Y*_2_, Entrapment Efficiency (%)
F1	1	15	738	53.51
F2	1	20	762	57.57
F3	1	30	774	59.20
F4	1.5	15	794	57.74
F5	1.5	20	828	62.20
F6	1.5	30	832	64.25
F7	2	15	836	68.39
F8	2	20	854	72.20
F9	2	30	859	74.12

**Table 3:** Evaluation parameters of *Punica granatum* ethosome gel.

Formulation	pH	Viscosity (CPS)	Spreadability (g.cm/s)	Grittiness
PGE1	7.1	34,015	35.15	No
PGE2	7.0	35,125	33.58	
PGE3	6.9	38,478	31.35	
PGE4	6.9	30,975	36.28	
PGE5	6.8	35,750	34.36	
PGE6	6.8	36,435	33.65	
PGE7	7.1	34,019	35.95	
PGE8	7.1	33,735	32.18	
PGE9	7.1	32,158	31.55	

**Table 4:** Stability studies of *P. granatum* ethosome gel.

Stability Study Period	Drug Content (%)	
Initial	4 ± 2 °C	98.5
	30 ± 2 °C	98.6
After 3 weeks	4 ± 2 °C	97.4
	30 ± 2 °C	96.7
After 6 weeks	4 ± 2 °C	97.2
	30 ± 2 °C	96.3
After 9 weeks	4 ± 2 °C	97.6
	30 ± 2 °C	96.3
After 12 weeks	4 ± 2 °C	97.5
	30 ± 2 °C	96.6

**Table 5:** Paw volume of different samples at different time intervals.

Groups	0 h	1 h	2 h	4 h	8 h	12 h
Control	0.0 ± 0	0.0 ± 0	0.0 ± 0	0.0 ± 0	0.0 ± 0	0.0 ± 0
Formalin	1.0 ± 0.01	1.6 ± 0.02	1.8 ± 0.03	1.9 ± 0.03	1.4 ± 0.02	1.2 ± 0.02
PG Extract	0.9 ± 0.01	1.4 ± 0.01	1.3 ± 0.01 *	1.2 ± 0.01 *	1.0 ± 0.01 *	0.9 ± 0.01 *
PG EthoGel	0.7 ± 0.01	0.9 ± 0.01 **	0.7 ± 0.01 **	0.6 ± 0.01 ***	0.5 ± 0.01 ***	0.5 ± 0.01 **
Standard	0.5 ± 0.01	0.7 ± 0.01 **	0.6 ± 0.01 ***	0.5 ± 0.01 ***	0.7 ± 0.01 **	0.6 ± 0.01 ***

One-way ANOVA followed by Tukey–Kramer multiple comparison test showed * *p* < 0.05, ** *p* < 0.01, and *** *p* < 0.00 as compared to the positive control group and the reference group.

**Table 6:** Anti-inflammatory activity of different samples on percentage inhibition of oedema.

Groups	0 h	1 h	3 h	5 h	12 h
Control	0.0%	0.0%	0.0%	0.0%	0.0%
PG EXTRACT	0.0%	24.6%	39.50%	46.13%	10.10%
PG ET GEL	0.0%	67.40%	74.10%	86.34%	82.64%
Standard	0.0%	71.20%	84.30%	70.12%	56.24%

**Table 7:** Development of ethosomes suspensions of methanolic extract of *P. granatum*.

Quantities in *w*/*w* % (100 gm)								
Code	EAP Extract	Carbapol 934 (% *w*/*v*) gms	Lecithin (*w*/*v*) (%)	Ethanol (*v*/*v*) mL	Cholesterol (%)	PEG 400 (*v*/*v*) mL	TEM (*w*/*v*) (%)	Water QS mL
EPG1	10	1.5	1	15	0.2	1	0.5	100
EPG2	10	1.5	1.5	15	0.2	1	0.5	100
EPG3	10	1.5	2	15	0.2	1	0.5	100
EPG4	10	1.5	1	20	0.2	1	0.5	100
EPG5	10	1.5	1.5	20	0.2	1	0.5	100
EPG6	10	1.5	2	20	0.2	1	0.5	100
EPG7	10	1.5	1	30	0.2	1	0.5	100
EPG8	10	1.5	1.5	30	0.2	1	0.5	100
EPG9	10	1.5	2	30	0.2	1	0.5	100

**Table 8:** Formulation of *P. granatum* ethosome.

Formulation Code	Lecithin (% *w*/*v*)	Ethanol (mL)	Cholesterol (%)
F1	1	15	0.2
F2	1	20	0.2
F3	1	30	0.2
F4	1.5	15	0.2
F5	1.5	20	0.2
F6	1.5	30	0.2
F7	2	15	0.2
F8	2	20	0.2
F9	2	30	0.2

## Data Availability

This study did not report any data.

## References

[1] Adu-Frimpong M., Firempong C.K., Omari-Siaw E., Wang Q., Mukhtar Y.M., Deng W., Yu Q., Xu X., Yu J. (2019). Preparation, optimization, and pharmacokinetic study of nanoliposomes loaded with triacylglycerol-bound punicic acid for increased antihepatotoxic activity. Drug Dev. Res..

[2] Middha S.K., Usha T., Pande V. (2013). A review on antihyperglycemic and antihepatoprotective activity of eco-friendly *Punica granatum* peel waste. Evid. Based Compl. Alt. Med..

[3] Khan J.A., Hanee S. (2011). Antibacterial properties of *Punica granatum* peels. Int. J. Appl. Biol. Pharm. Technol..

[4] Dkhil M.A. (2013). Anti-coccidial, anthelmintic and antioxidant activities of pomegranate (*Punica granatum*) peel extract. Parasitol. Res..

[5] Abdollahzadeh S., Mashouf R.Y., Moghaddam M.H., Roozbahani N., Vahedi M. (2011). Antibacterial and antifungal activities of *Punica granatum* peel extracts against oral pathogens. J. Dent..

[6] Devanesan S., AlSalhi M.S., Balaji R.V., Ranjitsingh A.J.A., Ahamed A., Alfuraydi A.A., AlQahatani F.Y., Aleanizy F.S., Othman A.H. (2018). Antimicrobial and cytotoxicity effects of synthesized silver nanoparticles from *Punica granatum* peel extract. Nanoscale Res. Lett..

[7] Modaeinama S., Abasi M., Abbasi M.M., Jahanban-Esfahlan R. (2015). Anti tumoral properties of *Punica granatum* (pomegranate) peel extract on different human cancer cells. Asian Pac. J. Cancer Prev..

[8] Moghaddam G., Sharifzadeh M., Hassanzadeh G., Khanavi M., Hajimahmoodi M. (2013). Anti-ulcerogenic activity of the pomegranate peel (*Punica granatum*) methanol extract. Food Nutr. Sci..

[9] Khan I., Rahman H., Abd El-Salam N.M., Tawab A., Hussain A., Khan T.A., Khan U.A., Qasim M., Adnan M., Azizullah A. (2017). *Punica granatum* peel extracts: HPLC fractionation and LC MS analysis to quest compounds having activity against multidrug resistant bacteria. BMC Complement Alt. Med..

[10] Wang D., Özen C., Abu-Reidah I.M., Chigurupati S., Patra J.K., Horbanczuk J.O., Jozwik A., Tzvetkov N.T., Uhrin P., Atanasov A.G. (2018). Vasculoprotective effects of pomegranate (*Punica granatum* L.). Front. Pharmacol..

[11] Aslam S., Jahan N., Khalil-Ur-Rehman, Ali S. (2019). Formulation, optimisation and in-vitro, in-vivo evaluation of surfactant stabilised nanosuspension of *Ginkgo biloba*. J. Microencapsul..

[12] Saraf S. (2010). Applications of novel drug delivery system for herbal formulations. Fitoterapia.

[13] Bailey M.M., Berkland C.J. (2009). Nanoparticle formulations in pulmonary drug delivery. Med. Res. Rev..

[14] Mahdi W.A., Alam P., Alshetaili A., Alshehri S., Ghoneim M.M., Shakeel F. (2022). Product development studies cranberry seed oil nanoemulsion. Processes.

[15] Shoaib A., Azmi L., Pal S., Alqahtani S.S., Rahamathulla M., Hani U., Alshehri S., Ghoneim M.M., Shakeel F. (2022). Integrating nanotechnology with naturally occurring phytochemicals in neuropathy induced by diabetes. J. Mol. Liq..

[16] Moradi S.Z., Momtaz S., Bayrami Z., Farzaei M.H., Abdollahi M. (2020). Nanoformulations of herbal extracts in treatment of neurodegenerative disorders. Front. Bioeng. Biotechnol..

[17] Khogta S., Patel J., Barve K., Londhe V. (2020). Herbal nano-formulations for topical delivery. J. Herbal Med..

[18] Wickramasinghe A.S.D., Kalansuriya P., Attanayake A.P. (2022). Nanoformulation of plant-based natural products for type 2 diabetes mellitus: From formulation design to therapeutic applications. Curr. Ther. Res..

[19] Jain P., Taleuzzaman M., Kala C., Kumar Gupta D., Ali A., Aslam M. (2021). Quality by design (Qbd) assisted development of phytosomal gel of *Aloe vera* extract for topical delivery. J. Liposome Res..

[20] Kaur J., Anwer M.K., Sartaj A., Panda B.P., Ali A., Zafar A., Kumar V., Gilani S.J., Kala C., Taleuzzaman M. (2022). ZnO Nanoparticles of *Rubia cordifolia* extract formulation developed and optimized with QbD application, considering ex vivo skin permeation, antimicrobial and antioxidant properties. Molecules.

[21] Mekjaruskul C., Sripanidkulchai B. (2019). *Kaempferia parviflora* nanosuspension formulation for scalability and improvement of dissolution profiles and intestinal absorption. AAPS PharmSciTech..

[22] Bose A., Wong T.W., Singh N. (2013). Formulation development and optimization of sustained release matrix tablet of Itopride HCl by response surface methodology and its evaluation of release kinetics. Saudi Pharm. J..

[23] Lee I.O., Jeong Y.S. (2002). Effects of different concentrations of formalin on paw edema and pain behaviors in rats. J. Korean Med. Sci..

[24] Németh Z., Pallagi E., Dobó D.G., Kozma G., Kónya Z., Csóka I. (2021). An updated risk assessment as part of the QbD-based liposome design and development. Pharmaceutics.

[25] Baboota S., Shakeel F., Kohli K. (2006). Formulation and evaluation of once-a-day transdermal gels of diclofenac diethylamine. Methods Find. Exp. Clin. Pharmacol..

[26] Wong Y.C., Ahmad-Mudzaqqir M.Y., Wan-Nurdiyana W.A. (2014). Extraction of essential oil from cinnamon (*Cinnamomum zeylanicum*). Oriental J. Chem..

[27] Barupal A.K., Gupta V., Ramteke S. (2010). Preparation and characterization of ethosomes for topical delivery of aceclofenac. Indian J. Pharm. Sci..

[28] Amarachinta P.R., Sharma G., Samed N., Chettupalli A.K., Alle M., Kim J.-C. (2021). Central composite design for the development of carvedilol-loaded transdermal ethosomal hydrogel for extended and enhanced anti-hypertensive effect. J. Nanobiotechnol..

[29] Khan P., Akhtar N. (2022). Phytochemical investigations and development of ethosomal gel with *Brassica oleraceae* L. (Brassicaceae) extract: An innovative nano approach towards cosmetic and pharmaceutical industry. Ind. Crops Prod..

[30] Kumar R., Mirza M.A., Naseef P.P., Kuruniyan M.S., Zakir F., Aggarwal G. (2022). Exploring the potential of natural product-based nanomedicine for maintaining oral health. Molecules.

[31] Sudhakar K., Mishra V., Jain S., Rompicherla N.C., Malviya N., Tambuwala M.M. (2021). Development and evaluation of the effect of ethanol and surfactant in vesicular carriers on lamivudine permeation through the skin. Int. J. Pharm..

[32] Graily-Moradi F., Asgari Lajayer B., Saglam N., Korkusuz F., Prasad R. (2021). Nanoinsecticides: Preparation, application, and mode of action. Nanotechnology Applications in Health and Environmental Sciences. Nanotechnology in the Life Sciences.

[33] Ghidan A.Y., Al-Antary T.M., Awwad A.M. (2016). Green synthesis of copper oxide nanoparticles using *Punica granatum* peels extract: Effect on green peach aphid. Env. Nanotechnol. Monit. Manag..

[34] He Y., Du Z., Lv H., Jia Q., Tang Z., Zheng X., Zhang K., Zhao F. (2013). Green synthesis of silver nanoparticles by *Chrysanthemum morifolium* Ramat. extract and their application in clinical ultrasound gel. Int. J. Nanomed..

[35] Gavan A., Colobatiu L., Hanganu D., Bogdan C., Olah N.K., Achim M., Mirel S. (2022). Development and evaluation of hydrogel wound dressings loaded with herbal extracts. Processes.

[36] Akhtar N., Akhtar N., Menaa F., Alharbi W., Alaryani F.S.S., Alqahtani A.M., Ahmad F. (2022). Fabrication of ethosomes containing tocopherol acetate to enhance transdermal permeation: In vitro and ex vivo characterizations. Gels.

[37] Salamanca C.H., Barrera-Ocampo A., Lasso J.C., Camacho N., Yarce C.J. (2018). Franz diffusion cell approach for pre-formulation characterisation of ketoprofen semi-solid dosage forms. Pharmaceutics.

[38] Yusefi M., Lee-Kiun Soon M., Teow S.Y., Monchouguy E.I., Neerooa B.N.H.M., Izadiyan Z., Jahangirian H., Rafiee-Moghaddam R., Webster T.J., Shameli K. (2022). Fabrication of cellulose nanocrystals as potential anticancer drug delivery systems for colorectal cancer treatment. Int. J. Biol. Macromol..

[39] Ajazuddin A.A., Khichariya A., Gupta S., Patel R.J., Giri T.K., Tripathi D.K. (2013). Recent expansions in an emergent novel drug delivery technology: Emulgel. J. Control. Release.

[40] Dave V., Bhardwaj N., Gupta N., Tak K. (2020). Herbal ethosomal gel containing luliconazole for productive relevance in the field of biomedicine. 3 Biotech..

[41] Bekhit A.A., Nasralla S.N., El-Agroudy E.J., Hamouda N., El-Fattah A.A., Bekhit S.A., Amagase K., Ibrahim T.M. (2022). Investigation of the anti-inflammatory and analgesic activities of promising pyrazole derivative. Eur. J. Pharm. Sci..

[42] Kumar T., Jain V. (2014). Antinociceptive and anti-Inflammatory activities of *Bridelia retusa* methanolic fruit extract in experimental animals. Scient. World J..

[43] Almeida D.S., da Silva D., Moreira L., Menegatti R., Lião L.M., Sanz G., Vaz B.G., Ghedini P.C., Costa E.A., Florentino I.F. (2020). Investigation of anti-inflammatory potential of 5-(3,5-di-tert-butyl-4-hydroxybenzylidene)-2-thioxodihydropyrimidine-4,6(1H,5H)-dione compound. Eur. J. Pharmacol..

[44] Aziz T.A., Kareem A.A., Othman H.H., Ahmed Z.A. (2020). The anti-inflammatory effect of different doses of aliskiren in rat models of inflammation. Drug Des. Dev. Ther..

[45] Eltom S.E.M., Abdellatif A.A.H., Maswadeh H., Al-Omar M.S., Abdel-Hafez A.A., Mohammed H.A., Agabein E.M., Alqasoomi I., Alrashidi S.A., Sajid M.S.M. (2021). The anti-Inflammatory effect of a γ-lactone isolated from ostrich oil of *Struthio camelus* (Ratite) and its formulated nano-emulsion in formalin-induced paw edema. Molecules.

